# Comparison of Misoprostol and Mefenamic Acid on Reducing Menstrual Bleeding in Patients Suffering From Heavy Menstrual Bleeding 

**Published:** 2019-09

**Authors:** Tahereh Eftekhar, Marjan Ghaemi, Aref Abedi, Mahboobeh Shirazi

**Affiliations:** 1 Reproductive Health Research Center, Tehran University of Medical Sciences, Tehran, Iran; 2 Fetal and Neonatal Research Center, Yas Hospital, Tehran University of Medical Sciences, Tehran, Iran

**Keywords:** Dysmenorrhea, Mefenamic Acid, Menorrhagia, Misoprostol

## Abstract

**Objective:** Heavy menstrual bleeding is one of the most frequent complaints of women. Various therapeutic approaches have been applied to treat this condition. In this study, we compared the efficacy of mefenamic acid and misoprostol in reducing menorrhagia.

**Materials and methods:** This is a randomized clinical trial study performed on 60 patients with menorrhagia. They were divided into two equal groups and randomly received mefenamic acid or misoprostol. Cycle duration, bleeding volume (according to the pictorial blood assessment chart), hemoglobin, hematocrit, and pad count were recorded before and after treatment. Side effects of treatment regimens were recorded.

**Results:** Blood loss volume per menstruation day in the mefenamic acid group was 118.40 ± 36.26 ml before treatment which decreased to 48.50 ± 24.71 ml after treatment (p = 0.262). Misoprostol reduced menstrual bleeding volume from 135.37 ± 34.85 ml per day to 49.40 ± 32.161 ml (p = 0.003). Mean duration of the menstrual period in patients receiveding mefenamic acid was 9.50 ± 3.27 days which decreased to 7.73 ± 2.14 days after treatment (p = 0.001). The similar change occurred in the misoprostol group and the mean duration of the menstrual period decreased from 7.70 ± 2.10 to 6.37 ± 2.29 days (p = 0.002). The number of pads used by patients in the mefenamic acid group before treatment was 23.20 ± 12.61 which was decreased to 14.33 ± 5.86 after treatment (p = 0.001). This alteration in misoprostol group was from 20.67 ± 6.12 to 15.53 ± 6.49 (p = 0.001).

**Conclusion:** Misoprostol can significantly reduce menstrual bleeding.

## Introduction

Heavy menstrual bleeding is a common complaint leading to poor quality of life and iron deficiency anemia in reproductive-aged women (1). Vaginal bleeding may have intra and extra-uterine causes. Dysfunctional bleeding is a term to describe a spectrum of abnormal menstrual bleeding patterns. It may occur in ovulatory women without medical disorders or pelvic pathologies (2). The normal cycle is defined as 28 days ± 7 days. Average volume of bleeding in a normal cycle is 35 milliliters (ml). Changes in duration and volume of bleeding lead to menorrhagia which occurs in any age due to anatomic abnormalities, pregnancy complications, hematologic disorders, infections, malignancies, drugs, obesity, systemic disorders and endocrine disturbances (2).

Heavy menstrual bleeding is defined as blood loss in a manner that interferes with physical, psychological and social quality of one’s life (3). An objective definition of heavy menstrual bleeding is blood loss of more than 80 ml in a normal menstrual cycle or bleeding more than 7 days (4). About 20-30% of women, experience heavy menstrual bleeding (5), but only 10 percent of these women suffered from heavy bleeding complications like anemia, therefore, some of them could not be included in clinical definition of menorrhagia (6). It is estimated that 60% of all secondary care referrals for menorrhagia resulted in hysterectomy (7).

Different etiologies necessitate specific treatment methods and through the years, a bunch of treatment strategies has been established. These treatments are divided into hormonal, non-hormonal and surgical modalities. Anti-fibrinolytic agents represent the main group of non-hormonal treatments while oral contraceptive pills, systemic progesterone, levonorgestrel-releasing intrauterine systems (LNG-IUS), and gonadotropin-releasing hormone (GnRH) analogs are the hormonal treatments. 

Prostaglandins increase vascular permeability and prevent platelets’ activity. So local prostaglandin release can increase endometrial bleeding (8). Due to different methods available in this field, considering the advantages and disadvantages of each treatment is so crucial to match the treatment modality for the patient’s condition (9).

 Misoprostol is a strong uterotonic agent with prominent vascular effects. Not only misoprostol has vasodilator effects in vascular beds but also has vasoconstriction effects in the limbs (10) and kidneys in human (11). It has been suggested that oral misoprostol has a strong vasoconstrictive activity on uterine arteries in the first hour after administration (12). Therefore it is hypnotized that this mechanism may reduce the menstrual blood flow. There is evidence from randomized controlled trials that misoprostol may reduce uterine bleeding during myomectomy (13). Only a few pieces of research are available about misoprostol in treating menorrhagia. The aim of this study is to compare misoprostol and mefenamic acid in reducing menstrual blood loss in women with menorrhagia.

## Materials and methods

This was a randomized clinical trial, a double-blind parallel design which was conducted in the gynecology clinic of Vali-e-Asr hospital, Tehran, Iran. The study was approved in Iranian registry of clinical trials (IRCT). The approval number is IRCT2014040912790N2. Sixty patients aging between 20 to 55 years old, complaining of menorrhagia were enrolled in this study. A complete history was taken from the patients, physical examination was performed and patient’s information and other findings recorded in a questionnaire. Cases with cardiac, vascular, metabolic, renal or hepatic disorders or taking any medication for menorrhagia, were excluded. Trans-vaginal ultrasonography examination was done to rule out ovarian tumors and endometrial hyperplasia. Laboratory tests including fasting blood sugar (FBS), β-subunit of human chorionic gonadotropin (βHCG), complete blood count (CBC), blood urea nitrogen (BUN), creatinine (Cr), thyroid-stimulating hormone (TSH), bleeding time (B.T), clotting time (C.T), prothrombin time (PT), serum glutamic oxaloacetic transaminase (SGOT), partial thromboplastin time (PTT), and serum glutamic pyruvic transaminase (SGPT) were done for ruling out other etiologies. Subjects were then randomly allocated into two separate groups (each group had 30 patients). One group took mefenamic acid capsules made in Iran, (Alhavi Pharmaceutical Company) and the second group took misoprostol tablets made in Iran, (Samisaz Pharmaceutical Company), distributed in completely identical packages. Patients were followed up for two consecutive cycles and blood loss volume was evaluated in the first and second cycles. In the two cycles, three doses of 250 mg mefenamic acid capsules or three doses of 200 µg misoprostol) tablets were taken after meal within 3 days. Pictorial blood assessment chart (PBAC) was used for assessing blood loss volume in patients. 

Data from questionnaires and PBAC chart were collected. Data were analyzed with SPSS 16 software. T-test and chi-square test were used. The study was approved by the board committee of obstetrics and gynecology of Tehran University of Medical Sciences. Written informed consent was taken from patients and they allowed to quit the study whenever they wanted.

## Results

Sixty participants (30 in each group) were enrolled in the trial. There was no significant difference between the two groups with regard to age, parity, body mass index (BMI), and endometrial thickness. Table 1 presents the demographic information of patients in the two groups.

**Table 1:** Demographic characteristics of the participants in each group

	**Mefenamic acid** **Mean (SD)**	**Misoprostol** **Mean (SD)**
Age(years)	39.57 (7.44)	37.97(8.63)
Height(meter)	1.61(0.04)	1.63(0.04)
Weight(kilogram)	70.47(9.96)	67.43(8.04)
BMI(kg/m^2^)	26.93(3.63)	25.21(3.52)

Mean duration of the menstrual period in patients received mefenamic acid was 9.50 ± 3.27 days which decreased to 7.73 ± 2.14 after treatment (p-value = 0.001). The similar change occurred in misoprostol group and the mean duration of the menstrual period decreased from 7.70 ± 2.10 to 6.37 ± 2.29 days (p-value = 0.002) so these two agents act effectively in reducing the duration of the menstrual period.

The number of pads used by patients in the mefenamic acid group before treatment was 23.20 ± 12.61 and lowered to 14.33 ± 5.86 after treatment (p-value = 0.001). This alteration in misoprostol group was from 20.67 ± 6.12 to 15.53± 6.49 (p-value = 0.001). It can be concluded that both medications are effective in lowering the number of the used pad.

The severity of dysmenorrhea in patients of mefenamic acid group decreased from 6.67 ± 1.83 to 3.62 ± 1.44 after treatment (p-value = 0.001) but this change in misoprostol group was from 5.60 ± 1.93 to 3.5 ± 1.79 (p-value = 0.40). It seems that misoprostol could not reduce the severity of dysmenorrhea significantly.

The blood indices were measured before and after treatment in both groups that can be seen in Table 2. Hemoglobin (Hb) and hematocrit (Hct) did not change significantly in both groups.

Blood loss volume in the mefenamic acid group was 118.4 ± 36.26 ml before treatment which decreased to 48.50 ± 24.71 ml after treatment (p-value = 0.262). Misoprostol could reduce menstrual bleeding volume from 135.37 ± 34.85 to 49.40± 32.161 ml (p-value = 0.003). 

The most prevalent side-effects in mefenamic acid and misoprostol groups were nausea and fever respectively. As seen in Table 3, there were no significant differences in side effects between the two groups. 

## Discussion

Excessive menstrual blood loss is an important healthcare problem. Effective medical treatment will improve the patients’ choice and provides an alternative to surgery. The cause of dysfunctional uterine bleeding (DUB) is thought to be at the level of the endometrium with several presumed abnormalities in the patient’s endometrium including increasing fibrinolytic activity and production of the prostaglandins (14). 

Various treatments have been proposed for heavy menstrual bleeding (15, 16). The use of misoprostol as a treatment to reduce menstrual blood loss (MBL) has been presumed, based on its potent stimulatory effect on the myometrium (8, 12).

The study of Ibrahim et al. over 60 women with heavy menstrual bleeding and regular periods without pelvic, hematologic and endocrine pathologies with short duration of menstrual bleeding and severe dysmenorrhea suggested that both rectal and oral misoprostol appear to be safe and reasonably effective routes for reducing bleeding in cases of menorrhagia but the rectal route is more effective. Their study showed that a significant blood loss reduction occurred in both oral and rectal misoprostol group (17).

We showed that mefenamic acid reduces the severity of dysmenorrhea effectively and misoprostol decreases menstrual bleeding volume significantly, but mefenamic acid could not reduce bleeding significantly. 

**Table 2:** Comparing blood indices before and after intervention in both groups

	**Mefenamic acid** **Mean (SD)**	**p-value**	**Misoprostol** **Mean (SD)**	**p-value**
Hb (before treatment)	12.04 (1.09)	0.443	12.02 (5.13)	0.13
Hb (after treatment)	13.36 (4.33)		11.93 (1.06)	
Hct (before treatment)	35.37 (3.77)	0.09	33.57 (5.31)	0.054
Hct (after treatment)	36.95 (6.35)		33.05 (3.19)	

**Table 3:** comparison of the side-effects between two groups

**Adverse effects**	**Mefenamic acid (Case)**	**Misoprostol (Case)**	**Total (Case)**	**p-value**
Nausea	5	3	8	0.70
Diarrhea	2	0	2	0.49
Vomiting	3	1	4	0.61
Fever	2	6	8	0.25

Mefenamic acid has been known as a prostaglandin synthesis inhibitor and is used for reducing blood loss volume. Fraser et al. conducted a trial on 69 women with menorrhagia and treated them with mefenamic acid. They showed a significant reduction in dysmenorrhea, headache, nausea, vomiting, depression, and the number of pads used. There was also a significant increase in ferritin levels (18). In our study, mefenamic acid could reduce blood loss volume up to 55 percent but this amount was not significant. Tranexamic acid as a member of this medication group has acted effectively in reducing heavy menstrual bleeding in another study (19).

Fraser and colleagues showed that the duration of the menstrual period was reduced with mefenamic acid significantly which is consistent with our findings (18). Bonnar et al. have reported that mefenamic acid could also reduce sanitary towels’ usage significantly (15), which is consistent with our results. Misoprostol could also reduce pad counts use in our study which is probably due to the reduction in blood loss volume. 

Kinitis et al. conducted a study on 35 patients with severe dysmenorrhea who were treated with mefenamic acid. After 3 cycles, 31 patients (88.6%) were completely free of pain, 2 patients reported a moderate reduction in pain while only 5 patients (13%) reported mild to moderate pain reduction in the placebo group (17). Our findings are consistent with this study that mefenamic acid could reduce dysmenorrhea significantly. 

Hb and Hct did not significantly change in the misoprostol group while it seems that a significant reduction in blood loss in this group should lead to elevations in these two indices. It seems that for detectable and significant changes in these two indices, longer follow-up is needed. Evaluation in a short-term period does not allow us to make accurate judgments. Concomitant use of misoprostol and mefenamic acid, the use of appropriate methods and doses of misoprostol for termination of pregnancy has been studied (20, 21). Concomitant use of misoprostol and mefenamic acid with appropriate doses to treat dysmenorrhea and rogue menorrhagia may be beneficial.

## Conclusion

Using misoprostol after complete examination for ruling out pregnancy can reduce menstrual bleeding efficiently. For reducing discomfort and pain, mefenamic acid is helpful. Further research is recommended to analyze the combined effects of these medications.
